# Qualitative Assessment of Contrast-Enhanced Ultrasound in Differentiating Clear Cell Renal Cell Carcinoma and Oncocytoma

**DOI:** 10.3390/jcm12093070

**Published:** 2023-04-23

**Authors:** Antonio Tufano, Costantino Leonardo, Chiara Di Bella, Giuseppe Lucarelli, Vincenzo Dolcetti, Piervito Dipinto, Flavia Proietti, Rocco Simone Flammia, Umberto Anceschi, Sisto Perdonà, Giorgio Franco, Alessandro Sciarra, Giovanni Battista Di Pierro, Vito Cantisani

**Affiliations:** 1 Department of Maternal-Child and Urological Sciences, Policlinico Umberto I Hospital, Sapienza University of Rome, 00162 Rome, Italy; 2 Department of Radiological Sciences, Oncology and Pathology, Sapienza University of Rome, 00162 Rome, Italy; 3 Department of Urology, IRCCS “Regina Elena” National Cancer Institute, 00144 Rome, Italy; 4 Fondazione “G. Pascale” IRCCS, 80131 Naples, Italy

**Keywords:** CEUS, contrast-enhanced ultrasound, kidney cancer, renal cancer, renal masses, kidney masses, accuracy, oncocytoma

## Abstract

Background: We aimed to assess whether clear cell renal cell carcinoma (ccRCC) can be differentiated from renal oncocytoma (RO) on a contrast-enhanced ultrasound (CEUS). Methods: Between January 2021 and October 2022, we retrospectively queried and analyzed our prospectively maintained dataset. Renal mass features were scrutinized with conventional ultrasound imaging (CUS) and CEUS. All lesions were confirmed by histopathologic diagnoses after nephron-sparing surgery (NSS). A multivariable analysis was performed to identify the potential predictors of ccRCC. The area under the curve (AUC) was depicted in order to assess the diagnostic accuracy of the multivariable model. Results: A total of 126 renal masses, including 103 (81.7%) ccRCC and 23 (18.3%) RO, matched our inclusion criteria. Among these two groups, we found significant differences in terms of enhancement (homogeneous vs. heterogeneous) (*p* < 0.001), wash-in (fast vs. synchronous/slow) (*p* = 0.004), wash-out (fast vs. synchronous/slow) (*p* = 0.001), and rim-like enhancement (*p* < 0.001). On the multivariate logistic regression, heterogeneous enhancement (OR: 19.37; *p* = <0.001) and rim-like enhancement (OR: 3.73; *p* = 0.049) were independent predictors of ccRCC. Finally, these two variables had an AUC of 82.5% and 75.3%, respectively. Conclusions: Diagnostic imaging for presurgical planning is crucial in the choice of either conservative or radical management. CEUS, with its unique features, revealed its usefulness in differentiating ccRCC from RO.

## 1. Introduction

Clear cell renal cell carcinoma (ccRCC) is the most common malignancy involving the kidney [1]. The majority of ccRCCs are incidentally detected by the use of modern imaging tools [2]. Contrast-enhanced CT (CECT) scanning is most often used for the assessment of renal masses. However, CECT scanning has limitations, including exposure to ionizing radiation, as well as the use of potentially nephrotoxic and immunogenic iodinated contrast, which may be problematic in patients who have some degree of chronic renal failure. In this regard, contrast-enhanced ultrasounds (CEUSs) using microbubble-based contrast agents have emerged as a safe and accurate imaging modality for the diagnostic work-up of renal masses [3,4,5,6]. Notably, the 2017 European Federation of Societies for Ultrasound in Medicine and Biology (EFSUMB)’s guidelines indicate CEUS as a promising fast and non-invasive method to discriminate between malignant and benign renal masses [7]. In this context, a relevant cohort of patients still undergoes renal surgery due to suspicious lesions that later histopathologically emerge as benign renal oncocytoma (RO) [8]. 

RO is well-defined epithelial tumor, representing from 3% to 7% of all renal neoplasms [9]. Its etiology remains poorly understood, although karyotypic translocations and aberrations have been described [10,11]. Interestingly, RO is not usually associated with an aggressive clinical course, showing an excellent prognosis at follow-up [12]. In terms of their gross appearance, the tumors are cortically localized, homogeneous, brown or tan, and well circumscribed [13,14]. Notably, RO tumors may exhibit, in their central region, a stellate appearance, commonly called a “fibrotic scar”, although their histologic appearance does not always correlate with this description since these scars are found in about 1/3 of cases [15]. Moreover, CECT and MRI imaging still present overlapping features among RO and ccRCC [16,17]. Indeed, non-invasive diagnoses of RO are still challenging due to the absence of biological serum markers and standardized imaging morphological characteristics.

Since clinical implications and therapeutic strategies may differ among ccRCC and RO, their preoperative identification would therefore be of great clinical interest. The aim of this study is to investigate the CEUS characteristics of ccRCC and RO. Moreover, our goal is to identify independent predictors of ccRCC from CEUS quantitative parameters.

## 2. Materials and Methods

### 2.1. Study Population

Between January 2021 and November 2022, we retrospectively queried and analyzed our prospectively maintained dataset. Inclusion criteria were as follows: (1) patients who had undergone conventional ultrasound imaging (CUS) and CEUS before nephron-sparing surgery (NSS); (2) patients who had undergone renal surgery with a final pathological specimen of ccRCC or RO; and (3) patients who had sufficient normal renal tissue around mass. Exclusion criteria were as follows: (1) age < 18 years; (2) pregnancy; (3) having a pure cystic lesion with no solid component; (4) being non-cooperative; and (5) having a general contraindication against performing a CEUS or being allergic to SonoVue (Bracco, Milan, Italy).

The study was conducted according to the principles of the Declaration of Helsinki and was approved by the institutional ethics committee (N.Prot. 0232/2020). Written informed consent was obtained from the individual participants before contrast-enhanced ultrasound (CEUS) was performed, and the patient health information (PHI) related to this study was protected. 

### 2.2. Imaging Acquisition and Interpretation

All baseline US and CEUS studies were performed with the same high-end US equipment (Samsung RS85, Samsung Medison, Seoul, South Korea) using 3.5–5.5 MHz convex probe. All CEUS examinations were analyzed by a single skilled radiologist (V.C.) with more than 15 years of clinical experience (EFSUMB Level 3). A 1–2.4 mL microbubble second-generation contrast agent (SonoVue, Bracco Imaging, Milan, Italy) was administered via intravenous injection, followed by 5–10 mL of 0.9% saline solution. Microbubble enhancement was performed continuously for at least 2 min. Although contrast phase terminology of the renal CEUS examination still remains controversial, in the present study, we relied on the following phase terms: cortical phases, which begin 10–15 s after injection until 30–45 s after the injection; and medullary phases, which begin approximately 30–45 s after injection until the microbubble echoes disappear completely [18]. 

According to EFSUMB guidelines, a real-time video clip was recorded for each patient and reviewed for the following: (I) CUS parameters (margins and echogenicity), (II) color Doppler flow imaging (CDFI) parameters (perilesional vs. mixed), and (III) CEUS qualitative parameters indicated as follows: (A) degree of enhancement indicated as higher than (hyper-enhancement), equal to (iso-enhancement), or less than (hypo-enhancement) that of the adjacent renal parenchyma after injection of contrast agent; (B) the homogeneity of enhancement (homogeneous vs. heterogeneous); (C) the presence of rim-like enhancement; (D) wash-in enhancement pattern, in which “fast-in”, “synchronous-in”, or “slow-in” indicated that the in-flow of the contrast agent from the tumors was faster than, simultaneous with, or slower than that from the adjacent cortex, respectively; (E) wash-out pattern, in which “fast-out”, “synchronous-out”, or “slow-out” indicated that the out-flow of the contrast agent from the tumors was faster than, simultaneous with, or slower than that from the adjacent cortex, respectively.

### 2.3. Statistical Analysis

All statistical analyses were performed using the Statistical Package for Social Sciences (SPSS) software v.26.0 (IBM Corp, Armonk, NY, USA). Data were expressed as mean (SD) or as median (IQR) for qualitative and quantitative data, respectively. Differences between groups were analyzed using the Mann–Whitney U test for continuous variables and Chi square or Fisher’s exact tests for categorical variables. Univariable and multivariable logistic regression analyses were used to identify independent predictors related to ccRCC with CEUS. Diagnostic accuracy of the multivariable model was evaluated using area under the curve (AUC). Two-tailed *p* values of <0.05 were considered statistically significant.

## 3. Results

### 3.1. Characteristics of the Included Patients

A total of 110 patients with 126 pathologically proven renal tumors matched our inclusion criteria. Overall, 103 cases of (81.7%) ccRCC and 23 cases of (18.3%) RO were diagnosed. The demographic and baseline characteristics are presented in Table 1. The patients were most frequently males (69.1%) with a median age of 64 years (IQR: 54–71). The median tumor size was 31 mm (IQR: 20–50) and the majority of the patients presented an exophytic pattern (65.9%). Overall, according to the available baseline characteristics, the patients with malignant lesions did not differ significantly from the patients with benign lesions except for their gender (*p* = 0.047).

### 3.2. Renal Mass Characteristics on CUS and CEUS

On the CUS, statistically significant differences between ccRCC and RO in terms of echogenicity were identified (*p* < 0.001). However, there were no significant differences between ccRCC and RO in terms of margins (*p* = 0.071) and blood flow signals on the CDFI (*p* = 0.254) (Table 2). 

**Figure 1:**
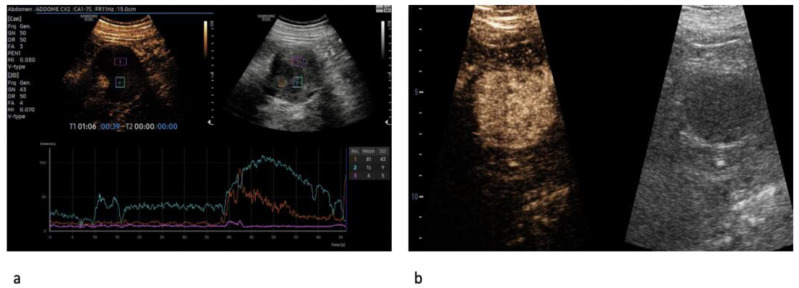
Heterogeneous enhancement pattern of clear cell renal cell carcinoma (ccRCC). The perfusion curves depict two different enhancement features for solid and cystic components, respectively (**a**). Homogeneous enhancement pattern in oncocytoma (**b**).

**Figure 2:**
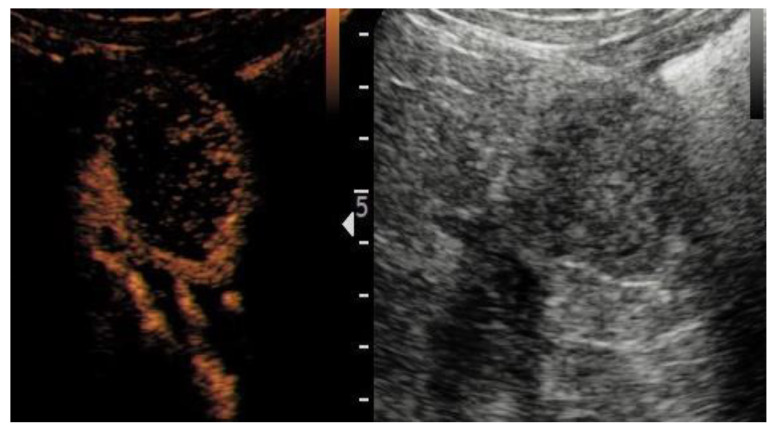
Rim enhancement pattern in clear cell renal cell carcinoma (ccRCC) with internal necrotic component.

On the CEUS, statistically significant differences between ccRCC and RO in terms of enhancement homogeneity were identified (*p* < 0.001). Specifically, patients with malignant lesions were more likely to exhibit a heterogeneous enhancement than patients with benign lesions (73.8% vs. 8.7%) (Figure 1a,b). Moreover, ccRCC cases were more likely to have a rapid contrast enhancement (wash-in) (80.6% vs. 52.2%; *p* = 0.004), a rapid wash-out (50.5% vs. 13.0%; *p* = 0.001), and the presence of rim-like enhancement (85.4% vs. 34.8%; *p* = <0.001) (Figure 2) compared to RO cases. 

### 3.3. Diagnostic Value of CEUS

The multivariate logistic regression analysis results showed that a heterogeneous enhancement (OR: 19.37; *p* = <0.001) and a rim-like enhancement (OR: 3.73; *p* = 0.049) were independent predictors of ccRCC (Table 3). 

**Figure 3:**
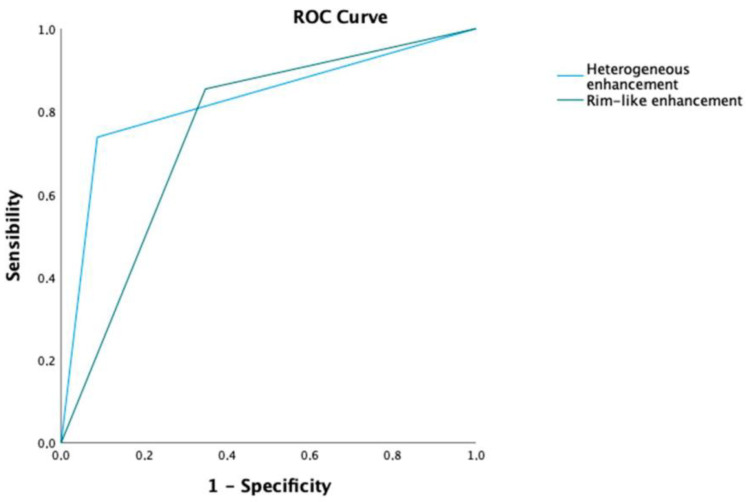
AUC of significant predictors of ccRCC in multivariable analysis.

Finally, the diagnostic accuracy for each significant finding of the multivariable analyses was evaluated. Here, heterogeneous enhancement and the presence of a rim-like enhancement had AUCs of 82.5% and 75.3%, respectively (Figure 3).

## 4. Discussion

In recent years, CEUSs have had significantly more applications across the urological community [19,20]. Notably, the perfusion process of kidney lesions with a CEUS is real-time, continuous, and dynamic. Therefore, the whole course of the contrast agent from the wash-in phase to the wash-out phase may be observed, representing an interesting additional advantage of CEUSs compared to CECTs and MRIs [21].

The aim of our study was supported by the EFSUMB guidelines, which state the need for further investigations to explore reliable diagnostic standards in this field [7]. The purpose of the present study, therefore, was to investigate the value of CEUSs in the differential diagnosis of ccRCC and RO. 

Our study led to several noteworthy findings. 

First, all lesions were managed with NSSs. According to the 2022 European Urology (EAU) guidelines, NSSs should be offered to patients with cT1 tumors, since a conservative kidney approach preserves renal function and potentially lowers the risk of cardiovascular disorders [22]. Interestingly, in our cohort, the median tumor size was 31 mm. Hence, our results may have important implications for the diagnostic workup of small renal masses (SRMs) (<4 cm). In a recent metanalysis addressing the accuracy of CEUSs in distinguishing benign vs. malignant SRMs, the CEUSs had an 89% accuracy rate [23]. However, only one study has investigated the accuracy of CEUSs for RO [18], in which all RO tumors were misdiagnosed by both the CEUSs and CT imaging. However, this report relied on a very limited sample size.

Second, significant differences in terms of echogenicity were reported among the ccRCC and RO tumors at CUS (*p* < 0.001). The first presented a more hypoechogenic pattern (60.2%), while RO tumors were more frequently isoechogenic (60.9%). However, the echotexture of ccRCC widely varies among studies [24,25]. Hence, depending solely on the echogenicity cannot reliably differentiate among benign and malignant renal masses. 

Third, in our cohort, a heterogeneous enhancement pattern on the CEUS was observed in 73.8% of the cases of ccRCC. In contrast, most RO (91.3%) cases exhibited a homogeneous enhancement. The multivariate logistic analysis confirmed heterogeneous enhancement as a strong predictive variable for detecting malignant masses (OR: 19.37; 95% CI: 3.37–111.45; *p* < 0.001). Notably, ccRCC frequently presents areas without enhancement, which usually correspond to intratumoral necrosis or cysts on histologic specimens [26]. Moreover, RO commonly appears on CEUSs with irregular, nonenhanced areas in the center due to the RO-related central stellate scar, although the histologic appearance does not always match this description [27]. Nevertheless, for small-size tumors, determining the true enhancement of kidney lesions using CEUSs remains challenging because of intrinsic limitations, such as pseudo-enhancements and the partial volume effect [28]. 

Fourth, we found that wash-out phase patterns (fast vs. synchronous/slow) were equally distributed among patients with ccRCC (50.5% vs. 49.5%). These data are in agreement with those of Xue et al., which also relied on a cohort with a similar sample size (n = 156) [20]. At the same time, RO more likely yielded a synchronous/slow wash-out (87%), which was higher than in the study presented by Schwarze et al. (50%) [29]. Notably, on CEUS imaging, the fast or synchronous wash-out mode indicates that the tumor has an arteriovenous shunt, while the delayed wash-out pattern could be related to a lack of vascular elastic fibers in tumor vessels, circuitous blood vessels within lesions, or a lack of an arteriovenous shunt, resulting in prolonged enhancement [30]. 

Fifth, rim-like enhancement, interpreted as the presence of a pseudocapsule, results from tumor growth, producing the compression, ischemia, and necrosis of adjacent normal parenchymas, with the consequent deposition of fibrous tissue [31,32]. In our study, the rim-like enhancement was confirmed to be a valid predictor of malignancy (O.R. 3.73; 95%CI 1.01–13.00; *p* = 0.049). Interestingly, this sign was found in 85.4% of our cohort, which is slightly greater than the percentage reported in the contemporary literature [33]. Moreover, previous studies have reported that the presence of a rim enhancement is a pathologic feature more often observed in the early stage of ccRCC or in low-grade ccRCC [34].

Taken together, whilst the use of CEUSs is well established in other clinical contexts, its use in kidney lesions is less recognized. In our study, CEUSs improve the diagnostic accuracy in detecting malignant renal masses and consequently potentiating the identification of benign lesions. For this reason, CEUSs could play a key role in helping detect findings which allow conservative treatments and warn against unnecessary surgery. Nevertheless, in the era of personalized medicine and big data, CEUS features can be applied for radiomics analyses in differentiating ccRCC from oncocytoma, as recently reported [35]. However, in order for this modality to be implemented in clinical practice, the standardization of image acquisition and segmentation protocols as well as the inter-institutional sharing of software are needed.

It should be acknowledged that there are several limitations in our study.

First, our study should be interpreted in the context of its retrospective and single-center design. Second, despite the qualitative CEUS parameters analyzed, residual confounding biases may have remained, e.g., we could not rely on quantitative variables. Specifically, no information regarding the time to peak, mean time to peak, and peak intensity was available. Third, our study is limited by the small sample size and relatively small percentage of benign tumors (18.3%). Fourth, the possibility of bias, resulting from the exclusion of patients undergoing active surveillance, should be considered. Nonetheless, all patients were diagnosed and treated at high-volume tertiary referral centers by experienced radiologists and uropathologists; hence, these findings may not be generalizable to all centers.

## 5. Conclusions

A CEUS, with its unique features, represents a useful adjunct imaging tool in the characterization and detection of ccRCC and RO. On our study, heterogeneous enhancement and rim-like enhancement were related to ccRCC. However, future prospective multicenter series to define indications as well as standardized qualitative and quantitative parameters are needed.

## Figures and Tables

**Table 1:** Demographic and baseline characteristics.

Variable	Overall n = 126	ccRCC n = 103	RO n = 23	*p* Value
Gender, n (%)				
Male	76 (69.1%)	60 (65.2%)	16 (88.9%)	0.047
Female	34 (30.9%)	32 (34.8%)	2 (11.1%)	
Age, years, median (IQR)	64 (54–71)	64 (54–71)	64 (55–74)	0.848
Laterality, n (%)				
Left	50 (48.1%)	40 (45.5%)	10 (62.5%)	0.209
Right	54 (51.9%)	48 (54.5%)	6 (37.5%)	
Location (%)				
Superior	50 (39.7%)	39 (37.9%)	11 (47.8%)	0.521
Middle	43 (34.1%)	35 (34.0%)	8 (34.8%)	
Inferior	33 (26.2%)	29 (28.1%)	4 (17.4%)	
Exophytic rate (%)				
>50% Exophytic	83 (65.9%)	67 (65.0%)	16 (69.6%)	0.68
>50% Endophytic	43 (34.1%)	36 (35.0%)	7 (30.4%)	
Size, mm (IQR)	31 (20–50)	32 (20–48)	30 (20–59)	0.924

ccRCC = clear cell renal cell cancer; RO = renal oncocytoma.

**Table 2:** Comparison of CUS and CEUS characteristics between malignant and benign renal tumors.

Variable	Overall n = 126	ccRCC n = 103	RO n = 23	*p* Value
Margins				
Regular	97 (77%)	76 (73.8%)	21 (91.3%)	0.071
Irregular	29 (23%)	27 (26.2%)	2 (8.7%)	
Echogenicity				
Hypo	69 (54.8%)	62 (60.2%)	7 (30.4%)	<0.001
Iso	36 (28.6%)	22 (21.4%)	14 (60.9%)	
Hyper	21 (16.7%)	19 (18.4%)	2 (8.7%)	
CDFI				
Perilesional	42 (33.3%)	32 (31.1%)	10 (43.5%)	0.254
Mixed	84 (66.7%)	71 (68.9%)	13 (56.5%)	
Wash-in				
Fast	95 (75.4%)	83 (80.6%)	12 (52.2%)	0.004
Synchronous/Slow	31 (24.6%)	20 (19.4%)	11 (47.8%)	
Enhancement				
Homogeneous	48 (38.1%)	27 (26.2%)	21 (91.3%)	<0.001
Heterogeneous	78 (61.9%)	76 (73.8%)	2 (8.7%)	
Wash-out				
Fast	55 (43.7%)	52 (50.5%)	3 (13.0%)	0.001
Synchronous/Slow	71 (56.3%)	51 (49.5%)	20 (87.0%)	
Enhancement intensity				
Hyper	86 (68.3%)	68 (66%)	18 (78.3%)	
Iso/Hypo	40 (31.7%)	35 (34%)	5 (21.7%)	0.254
Rim-like enhancement				
No	30 (23.8%)	15 (14.6%)	15 (65.2%)	
Yes	96 (76.2%)	88 (85.4%)	8 (34.8%)	<0.001

ccRCC = clear cell renal cell cancer; RO = renal oncocytoma; CDFI = color Doppler flow imaging.

**Table 3:** Univariable and multivariable logistic regression analysis of CEUS characteristics to identify predictors of ccRCC.

Univariable Analysis				Multivariable Analysis		
Variable	O.R.	95% CI	*p* Value	O.R.	95% CI	*p* Value
Wash-in (Fast vs. Synchronous/Slow)	0.26	0.10–0.68	0.006	2.67	0.63–11.36	0.184
Enhancement (Heterogeneous vs. Homogeneous)	29.56	6.49–134.52	0.001	19.37	3.37–111.45	<0.001
Wash-out (Fast vs. Synchronous/Slow)	6.79	1.90–24.29	0.003	1.25	0.22–7.11	0.802
Enhancement intensity (Hyper vs. Iso/Hypo)	0.54	0.18–1.58	0.259	-	-	-
Rim-like enhancement	11.00	3.97–30.44	<0.001	3.73	1.01–13.00	0.049

## Data Availability

The data presented in this study are available on request from the corresponding author. The data are not publicly available due to ethical restrictions.
